# Loneliness in older persons with schizophrenia

**DOI:** 10.1177/00207640241307842

**Published:** 2024-12-23

**Authors:** Tess Patterson, Fatemeh Sajjadi, Linda Hobbs, Yoram Barak

**Affiliations:** 1 Department of Psychological Medicine, Dunedin School of Medicine, University of Otago, New Zealand; 2 Optentia Research Focus Area, North-West University, Potchefstroom, South Africa

**Keywords:** interRAI, schizophrenia, older adults, loneliness

## Abstract

**Background:**

In tandem with the rise in numbers of older adults in the general population, more people with schizophrenia (PwS) are also living longer. This vulnerable population has several trajectories of ageing driven by a number of social determinants of health, including the experience of loneliness and they may be more at risk of experiencing loneliness.

**Aim:**

This study aimed to examine demographic, psychosocial and clinical variables and their relative contribution to the loneliness of older PwS (OPwS) in a large New Zealand community sample.

**Method:**

New Zealanders 65 years and older who completed their first interRAI assessment during the study period were included. Data from 1,883 OPwS participants was analysed [mean age, 75.1 + 7.7 years; 1,132 (60.3%) females]. The majority were of European ethnicity (64.8%; Māori 15.7%, Pacifica 5.7%) and only a minority were married (20.6%). Chi-square analysis was used to examine relationships between loneliness and demographic and psychosocial variables. Logistic regression was used to measure the relative contribution of these variables to loneliness.

**Results:**

Being lonely was reported for 25.9% of OPwS, a significantly higher rate than that reported in the general population of people over 65 years-of-age. A relationship with loneliness was found for marital status, depression and living arrangements but not gender, ethnicity or social engagement. Co-morbid depression and not being in a marital-type partnership were identified as significant predictors of being lonely. Conversely, living with someone predicted being not lonely.

**Conclusions:**

Older community dwelling PwS experience higher rates of loneliness than older adults in a general population. Addressing loneliness, as well as its correlates, co-morbid depressive symptoms and living arrangements, is crucial to supporting the wellbeing of OPwS.

## Introduction

Loneliness, often conceptualized as social isolation, refers to a subjective emotional experience of a discrepancy between desired social relationships and perceived inadequate social relationships (Ong et al., 2016). It has been shown that loneliness is a significant risk factor for morbidity and mortality rates across the adult lifespan, even after controlling for biological, social and psychological risk factors (Kuiper et al., 2015; Lee et al., 2019; Margrett et al., 2011, Nicolaisen & Thorsen, 2014). Individuals with schizophrenia may be especially prone to loneliness due to being subject to stigma (Dickerson et al., 2002), impaired social cognition (Shah et al., 2012), experiencing higher rates of social challenges (Muralidharan et al., 2019), struggling to maintain functioning relationships (Bengtsson-Tops & Hansson, 2001) and limited social contact (Abdallah et al., 2009). While it is traditionally accepted that loneliness may be the negative consequence of psychotic symptoms, more recent perspectives suggest that loneliness may be part of a vicious cycle that contributes to the development of psychotic symptoms (Michalska da Rocha et al., 2018; van der Werf et al., 2010).

Loneliness is a public health threat to people with schizophrenia (PwS). Specifically, loneliness and social isolation predict an increased premature mortality risk, and the World Health Organization now lists ‘social support networks’ as a ‘determinant of health’. Loneliness is linked to cardiovascular disease and stroke (Valtorta et al., 2016) and to mortality. A meta-analysis including 70 studies encompassing 3.4 million people found that loneliness, social isolation and living alone all had a significant effect on the risk of dying prematurely with 29%, 26% and 32% increased likelihood of mortality respectively (Holt-Lunstad et al., 2015). Overall, loneliness appears to be distinct from other schizophrenia-related deficits, with loneliness having similar effects in patients with schizophrenia and non-psychiatric comparison subjects. This suggests that models of loneliness developed in the general population may generalize to schizophrenia (Eglit et al., 2018).

Schizophrenia is ranked third in causes of disability-adjusted life years among individuals aged 60 years and older (Cohen et al., 2015) and ageing with schizophrenia is associated with greater physical morbidity and increased mortality compared with control populations (Bersani et al., 2019; Shivakumar et al., 2014). As the number of older geropsychiatric patients is increasing, there is also a corresponding increase in OPwS (Taube et al., 2024). Understanding the biopsychosocial health needs of OPwS, especially in relation to loneliness, is essential to providing responsive care systems to reduce the poor health determinants that are related to loneliness. Prior research has identified that 21% of older adults in New Zealand (NZ) report feeling lonely (Jamieson et al., 2018), however, our understanding in respect to the rate of loneliness among OPwS is not documented. Given that 80% of OPwS live at home, with only 5% to 8% receiving care in facilities (Klug et al., 2006), there are potentially barriers to finding the relevant data needed to understand loneliness for OPwS. However, in NZ, home-based international Resident Assessment Instrument (interRAI) assessments are undertaken for all people, 65 years or older with functional impairment. As the interRAI dataset is comprehensive and validated (Nishtala & Jamieson, 2017) it provides us with an opportunity to examine data on loneliness in relation to OPwS.

The present study aims to analyse NZ interRAI data for OPwS living in the community to examine demographic, psychosocial and clinical variables and their relative contribution to loneliness. Specifically, this study (1) provides descriptive data for study variables (gender, age, ethnicity, loneliness, social engagement, marital status, depression, living arrangements) for the population of OPwS, (2) compares rates of loneliness in the OPwS group with data from the general population of older adults, (3) explores associations between loneliness and demographic data (gender, ethnicity and marital status) and psychosocial variables (depression, living arrangements and social engagement) and (4) examines whether any of these variables have predictive value for loneliness in OPwS.

## Methods

The international Resident Assessment Instrument (interRAI; www.interrai.co.nz) is a comprehensive evidence-based clinical assessment tool that is used worldwide to build a picture of a person’s health and wellbeing needs (Hogeveen et al., 2017). The 236-item electronically recorded assessment includes physical, psychological and cognitive domains, diagnoses including schizophrenia, and is administered by a certified healthcare professional when a person requires a clinical or social support care plan assessment.

In the current study, data were extracted from interRAI assessments conducted with people 65 years and older who had undergone their first interRAI assessment between September 2012 and September 2022 at any District Health Board (DHB, now Te Whatu Ora) across NZ. The data were completely anonymized; no identifying data (name, address or National Health Index number) were included. The data were included from those who (1) provided consent for their anonymized data to be used for research purposes; (2) were aged 65 years and older; (3) received a formal diagnosis of schizophrenia and (4) were living independently in the community (home, apartment, rented room). All diagnoses of schizophrenia are based on notification from a General Practitioner (GP), clinical specialist or from a hospital discharge summary (Mentzel et al., 2023). Only the initial interRAI assessment for each person was reviewed, even if an individual underwent subsequent assessments.

### Compliance with ethical standards

Ethical approval for this study was obtained from the University of Otago Ethics Committee, approval #HD22/078.

### Measurement

The interRAI is a suite of selected evidence-based clinical assessments that together forms a 236-item clinical assessment tool developed to assess a wide range of clinical and social aspects of an older person’s life including physical, psychological and cognitive domains. A person’s responses are used to determine which services best meet that person’s need. In NZ, the interRAI assessment, which has been mandatory since 2013, is a prerequisite for older adults who needs additional support services in their home or need to transfer to residential care (Barak, Leitch, & Glue, 2021). In this study, we analyse eight domains captured by the interRAI assessment, including demographic data (age, gender, marital status and ethnicity) and psychosocial domains that are associated with aging (depression, social engagement, loneliness and living arrangements).

### Depression

We used data from the Depression Rating Scale (DRS; Burrows et al., 2000) section of the interRAI assessment, to measure possible depressed, anxious and sad mood. We included seven indicators of depressed mood (observed in the last 3 days) each coded using a 3-point scale (0 = not present in last 3 days to 2 = exhibited daily in last 3 days). Similar to previous studies (Leitch et al., 2018) we calculated a total depression score and then categorized this score into one of three groups as follows, (1) no depression (DRS total score of 0), (2) mild to moderate depression (DRS total scores of 1–7) or (3) moderate to severe depression (DRS total scores of 8–14; Huang & Carpenter 2011).

### Social engagement

Social engagement was evaluated using responses to the following three interRAI items regarding participation in social activities: ‘Participation in social activities of long-standing interest’, ‘Visit with a long-standing social relation or family member’ and ‘Other interaction with long-standing social relation or family member such as phone or email. . .’ Clinicians code for these items using a 5-point scale (0 = never, 1 = more than 30 days ago, 2 = 8–30 days ago, 3 = 4–7 days ago and 4 = in the last 3 days). Similar to previous studies (Barak, Barson, et al., 2021) we calculated a total social engagement score and then dichotomized this score into (1) no-to-low social engagement (total score of 0–6) or (2) moderate-to-high levels of social engagement (total score of 7–12).

### Loneliness

Loneliness was evaluated using response to the interRAI psychosocial well-being item: ‘Says or indicates that she or he feels lonely’. Responses are recorded by the interRAI interviewer dichotomously as feeling: (1) lonely or (2) not feeling lonely.

### Living arrangements

We described living arrangements using interRAI data that recorded whether the client lives with another person, an ‘informal helper’ such as a spouse or relative and for how long (0 = no does not live with anyone, 1 = yes, has lived with someone for 6 months or less, 2 = yes, has lived with someone for more than 6 months and 8 = no informal helper). We dichotomized these responses to (1) lives alone (responses 0 = no does not live with anyone and 8 = no informal helper) or (2) not alone (i.e. lives with another person), (responses 1 = yes, 6 months or less and 2 = yes, more than 6 months).

### Marital status

We described marital status as it was recorded by the interRAI as being (1) widowed, separated or divorced, (2) never married, (3) married or (4) other.

### Statistical analysis

Descriptive statistics are presented for all study variables (gender, age, ethnicity, loneliness, social engagement, marital status, depression, living arrangements). Bivariate analyses (chi-square) were conducted to explore associations between the proportion of OPwS reported as lonely or not lonely with demographic data (i.e. gender, ethnicity and marital status), data from the general population of older adults (as reported by Leitch et al., 2018) and psychosocial variables (i.e. depression, living arrangement and social engagement). Post hoc analysis with Bonferroni correction for type 1 error were used to further evaluate pairwise relationships. Cramer’s *V* measures were included to present effect sizes. To further examine the possible effect of study variables on loneliness, we performed a binomial logistic regression analysis. We analysed the data using IBM SPSS Statistics 25 (IBM, 2017). For all the analyses, unless otherwise stated, statistical significance was determined by (two-sided when appropriate) *p* < .05.

## Results

Between September 2012 and September 2022, 2,620 interRAI first assessments were completed for people with schizophrenia (PwS) across District Health Boards (DHBs, now Te Whatu Ora) in NZ of which 1,883 (71.9%) assessments were for PwS 65 years and older, therefore 1,883 distinct OPwS were included. The analysed sample (mean (*M)* age = 75.05 years; standard deviation (*SD)* = 7.74; range: 65–102 years) consisted of 1,132 (60.0%) assessments for females and 745 (39.7%) for males (gender was not reported for six clients). Females (*M* age = 76.04; *SD* = 7.78) were significantly older than males (*M* age = 73.52; *SD* = 7.44; *t* (1,875) = 6.99, *p* < .001, Cohen’s *d* = 0.33). Interviewees identified mostly as NZ Europeans (1,221; 64.8%), Māori (295; 15.7%), Pacific peoples (107; 5.7%) and other ethnicities (260; 13.8%).

### Compared to general population of older adults

In this sample, 1,396 (74.1%) OPwS were recorded as not lonely, and 487 (25.9%) were recorded as lonely. To compare rates of loneliness recorded for OPwS with rates of loneliness recorded for the general population of older adults (aged 65 years and over), we used comparative data reported by Leitch et al. (2018) on the same loneliness measure extracted from interRAI. The rate of being recorded as lonely for OPwS (25.9%) was significantly greater than that for the general population of older adults (20.3%; 2 (loneliness: lonely, not lonely) × 2 (population: general older adult, OPwS) *X*^2^ (1, *N* = 75,133) = 35.29, *p* ⩽ .001).

### Relationships between loneliness and demographic or psychosocial factors

To investigate possible relationships between loneliness and demographic or psychosocial factors among OPwS we conducted a series of chi-square analyses, see Table 1.

**Table 1.** Psychosocial and demographic factors associated with loneliness.

Factor	Loneliness		Test	Cramer’s *V*
	Lonely (*n* = 487)	Not lonely (*n* = 1,396)		
Age (years)				
Mean (±*SD*)	74.88 (7.86)	75.11 (7.70)	*t* = 0.55, *p* > .05	
Range	65–102	65–102		
Gender				
Male	176 (36.3%)	569 (40.9%)	χ^2^(1) = 3.16, *p* > .05	.04
Female	309 (63.7%)	823 (59.1%)		
Ethnicity				
NZ European	317 (65.1%)	904 (64.8%)	χ^2^(3) = 2.07, *p* > .05	.03
Māori	83 (17.0%)	212 (15.2%)		
Pacifica	23 (4.7%)	84 (6.0%)		
Other ethnicities	64 (13.1%)	196 (14.0%)		
Marital status				
Never married	110 (22.7%)	385 (27.7%)	χ^2^(3) = 10.91*	.08
Married	89 (18.4%)	298 (21.4%)		
Widowed/separated/divorced	271 (55.9%)	657 (47.2%)		
Other	15 (3.1%)	52 (3.7%)		
Missing (excluded from χ^2^ analysis)	2	4		
Depression				
None	236 (48.5%)	937 (67.1%)	χ^2^(2) = 54**	.17
Mild-moderate	232 (47.6%)	430 (30.8%)		
Moderate-severe	19 (3.9%)	29 (2.1%)		
Living arrangement				
Not-alone (i.e. with someone)	138 (28.4%)	489 (35.0%)	χ^2^(1) = 7.14**	.06
Alone	348 (71.6%)	907 (65.0%)		
Missing (excluded from χ^2^ analysis)	1	–		
Social engagement				
No-low social engagement	270 (60.1%)	687 (56.2%)	χ^2^ (1) = 2.10, *p* > .05	.03
Moderate-high social engagement	179 (39.9%)	536 (43.8%)		
Missing (excluded from χ^2^ analysis)	38	173		

* *p* < .05. **p < .01 (two-tailed).

#### Gender and ethnicity

There was no significant association between gender and loneliness (2 (loneliness: lonely, not lonely) × 2 (gender: male, female) χ^2^(1, *N* = 1,877) = 3.16, *p* > .05; *C*_v_ = 0.04). A similar number of females recorded as lonely (27.3%) compared to males (23.6%). There was no relationship between loneliness and ethnicity (2 (loneliness: lonely, not lonely) × 4 (ethnicity: NZ European, Māori, Pacifica and other ethnicities) χ^2^(3, *N* = 1,883) = 2.07, *p* > .05; *C*_v_ = 0.03).

#### Social engagement

Half of the OPwS (957; 50.8%) were recorded as having no-to-low social engagement, 715 (38%) were recorded as having moderate-to-high levels of social engagement and for 211 (11.2%) a response was unable to be determined. There was no relationship between loneliness and social engagement (2 (loneliness: lonely, not lonely) × 2 (social engagement: no-low, moderate-high social engagement) χ^2^(1, *N* = 1,672) = 2.10, Fisher’s Exact *p* = .16; *C*_v_ = 0.03).

#### Marital status

Nearly half of the OPwS were recorded as widowed, separated or divorced (928; 49.3%), a smaller number as never married (495; 26.3%) and a minority as married (387; 20.6%). Marital status was not recorded for 73 clients. There was a significant association between loneliness and marital status (2 (loneliness: lonely, not lonely) × 4 (marital status: never married, married, widowed/separated/divorced, other) χ^2^(3, *N* = 1,877) = 10.91, *p* = .01; *C*_v_ = 0.08). Those OPwS who were recorded as having never married were more likely to be not lonely (27.7%) compared to lonely (22.7%) however accounting for type 1 error there was no significant difference (post-hoc *p* = .03, Bonferroni corrected criterion *p* = .006). Post hoc analysis showed those recorded as widowed, separated or divorced were significantly more likely to be lonely (55.9%) compared to not lonely (47.2%, post-hoc *p* = .001, Bonferroni corrected criterion *p* = .006). There were no differences for those recorded as married or those that were recorded as having an ‘other’ marital status. OPwS who were recorded as widowed, separated or divorced contributed the most to loneliness (standardized residual of 2).

#### Depression

Most of the OPwS (1,173; 62.3%) were recorded as having no depressive symptoms, 662 (35.2%) were recorded as having mild to moderate symptoms and 48 (2.5%) were recorded as having moderate to severe depressed mood and symptoms. There was a significant association between loneliness and depression (2 (loneliness: lonely, not lonely) × 3 (depression: none, mild-moderate, moderate-severe) χ^2^(2, *N* = 1,883) = 54, *p* < .001; *C*_v_ = 0.17). Post hoc analysis showed those with no depressive symptoms recorded were significantly more likely to be not lonely (67.1%) compared to lonely (48.5%, post-hoc *p* < .001, Bonferroni corrected criterion *p* = .008) and those with low-moderate depressive symptoms recorded were significantly more likely to be lonely (47.6%) compared to not lonely (30.8%, post-hoc *p* < .001, Bonferroni corrected criterion *p* = .008). Those with moderate-severe depressive symptoms recorded were also more likely to be lonely (3.9%) compared to not lonely (2.1%) however post hoc analysis accounting for Type 1 error showed no significant difference (post-hoc *p* = .03, Bonferroni corrected criterion *p* = .008). Those with mild-moderate depressive symptoms (with standardized residual of 4.6) were contributing the most in loneliness.

#### Living arrangements

A total of 627 (33.3%) OPwS were recorded as living with an informal helper, and 1,255 (66.6%) were recorded as living alone. There was a significant association between loneliness and living arrangements (2 (loneliness: lonely, not lonely) × 2 (living arrangements: lives alone, lives with someone) χ^2^(1, *N* = 1,882) = 7.14, *p* = .008; *C*_v_ = 0.06). Post hoc analysis shows that those recorded as living alone were more likely to be lonely (71.6%) compared to not lonely (65.0%, post-hoc *p* = .008, Bonferroni corrected criterion *p* = .013) and those recorded as living with someone were more likely to be not lonely (35.0%) compared to lonely (28.4%, post-hoc *p* = .008, Bonferroni corrected criterion *p* = .013).

### Predictors of loneliness

To determine whether any of the psychosocial factors examined here could differentiate between OPwS who were recorded as lonely and recorded as not lonely, we performed a binomial logistic regression analysis using only those independent variables that showed a significant effect in the previous chi-square analyses. That is, we used depression (none, mild-moderate or moderate-severe), marital status (never married, married, widowed/separated/divorced or other) and living arrangements (lives alone or not-alone) as the predictor variables with loneliness (lonely or not lonely) as the dependent variable. VIF scores were all below 10 indicating absence of multicollinearity of the independent variables. Overall, the logistical regression model was statistically significant (χ^2^(6) = 73.69, *p* < .001). The model explained 5.6% (Nagelkerke *R*^2^) of the variance in loneliness and correctly classified 74.2% of cases. For marital status, being widowed/separated/divorced (*B* = 0.43, *p* < .001, Wald = 10.41, Exp(*B*) = 1.54, 95% CI [1.18, 2.00]) was the strongest predictor of being lonely. For living arrangements, living with someone was associated with being not lonely (*B* = −0.36, *p* = .008, Wald = 6.59, Exp(*B*) = 0.701, 95% CI [0.54, 0.91]). We also found that low to moderate depression (*B* = 0.79, *p* < .001, Wald = 50.88, Exp(*B*) = 2.20, 95% CI [1.77, 2.73]) and moderate to severe depression (*B* = 1.01, *p* = .001, Wald = 10.79, Exp(*B*) = 2.76, 95% CI [1.51, 5.04]) predicted a high probability of being lonely. To further investigate the role of depression in predicting loneliness, we conducted a second logistic regression to examine the potential effect size of having ‘any’ depressive symptomology. We recategorized depression from three categories to a binomial measure, that is, depressive symptoms recorded as present or not present. We found that OPwS who reported some degree of depressive symptoms were 2.23 (95% CI [1.81, 2.76]) more likely to be recorded as lonely than OPwS who reported no depressive symptoms (*B* = 0.80, *p* < .001, Wald = 55.07, Exp(*B*) = 2.23, 95% CI [1.81, 2.76]).

## Discussion

There is increasing interest internationally in promoting active social lives in older age and counteracting pathways and outcomes related to social exclusion and loneliness for men and women in later life (Urbaniak et al., 2023). Loneliness, among older adults, is a risk factor for developing dementia, stroke, cancer and possibly increased risk of suicide (Dabiri et al., 2023). However, our understanding of the neurobiological and psychological pathways that link loneliness to adverse physical and psychological outcomes still remains limited and requires further exploration.

The present study examined loneliness and its’ determinants in OPwS from interRAI assessments undertaken in a large sample of community dwelling older people. One notable finding of the present study is that OPwS are lonelier than older adults in the general population. Also of note, psychosocial factors and mental health contributed to loneliness. That is, having depressive symptoms, being widowed, separated or divorced and living alone predicted loneliness in OPwS. Among these, the strongest predictor of loneliness for OPwS was being widowed, separated or divorced. Given the impact of severe mental illness on marriage and durability of relationships wherein individuals with mental disorders are at greater risk of marital dissolution (Mojtabai et al., 2017), this puts OPwS at an increased risk of loneliness. Furthermore, as living with someone predicted not being lonely but PwS have difficulty maintaining or developing social relationships and have limited contact with family (Abdallah et al., 2009; Bengtsson-Tops & Hansson, 2001), there is an increased likelihood that OPwS will live alone. In fact, in the present study we found that the majority of OPwS did live alone. In this way it is not surprising then that OPwS experience higher rates of loneliness and are at greater risk of experiencing loneliness. Given the impacts of loneliness on biopsychosocial health outcomes, loneliness becomes a key target in terms of clinical treatment need and intervention.

The present study also found that those who experienced depressive symptomatology were at more than twice the risk of experiencing loneliness in comparison to those OPwS who did not experience depressive symptomatology. Given that depression is commonly reported amongst OPwS (Cohen & Ryu, 2015; Diwan et al., 2007; Solomon et al., 2021) and the level of depression among older outpatients with schizophrenia is approximately one and a half times the level in the general older population (Cohen et al., 1996), this places OPwS more at risk of experiencing depression and based on the findings of the present study, more at risk of experiencing loneliness. Nearly 30 years ago, Cohen and colleagues attempted to develop a multifactorial model of predictors of depression among aging persons with schizophrenia living in the community. They found that depression was associated with diminished social network connections and fewer supportive social network members who provided sustenance such as food and money. These authors concluded that several of the variables associated with depression amongst OPwS are potentially alterable and therefore afford opportunities to enhance well-being (Cohen et al., 1996). However, in the succeeding years there has been limited research focussing on the complex interactions of depressive symptoms, ageing, schizophrenia and social determinants of health with instead, most publications focussing on pharmacological treatment options (Kasckow & Zisook, 2008). Although pharmacological agents are important in treatment, pharmacological agents in combination with psychosocial interventions are seen as important treatments for OPwS presenting with depressive symptoms (Felmet et al., 2011). More research combining pharmacologic and psychosocial interventions is needed to better understand how to treat this population of elderly patients. This is especially pertinent given the findings of the present study that depressive symptoms predict higher rates of loneliness in OPwS, and higher rates of loneliness are associated with morbidity.

In considering interventions for loneliness, it is important to note that loneliness is considered a multi-faceted construct with varying implications for individuals with psychotic symptoms (Cohen, 2022). On the one hand, Cacioppo and Cacioppo (2018) argue that loneliness has an adaptive process that promotes individuals to engage more in social activities and rebuild their network. This argument is supported by evidence suggesting that lonely individuals with psychotic symptoms use healthcare services 2.6 times more than non-lonely individuals (Badcock et al., 2020), which reflects their need for social engagement. Similarly, Cohen (2022) suggests that while social networks may decline at illness onset, later life may be a time of rebuilding social networks and connections. Increasing social interaction may therefore be beneficial to reduce the prevalence of loneliness among older adults (Domènech-Abella et al., 2017). On the other hand, Chrostek et al. (2016) argue that seeking social connections may serve as a maladaptive process that leads to social withdrawal and a person feeling even more lonely. That is, feelings of loneliness may lead to seeking social interactions, and stigmatizing attitudes associated with the social interactions may worsen loneliness symptoms. These contrasting views on adaptive versus maladaptive processes related to loneliness indicate the need for care and further research examining social and behavioural interactions and the effect they have for OPwS. The findings of the present study indicate interventions are needed to target loneliness amongst OPwS, for example targeting barriers, such as poor social networks and impaired interpersonal skills (Lim et al., 2018) or increasing opportunities for social interactions.

### Limitations

The present study has the following limitations: The interRAI assessment inherently results in a biased sample of older adults who are frailer than the general older population (Ludwig & Busnel, 2017). Data reported was collected over a 10-year period that included the COVID-19 pandemic that may have impacted experiences of loneliness amongst OPwS. The dichotomous capture of loneliness by the interRAI measure does not allow for a more holistic definition of loneliness and may not have captured the more nuanced experiences of OPwS. Similarly, the use of the Depression Rating Scale (DRS) has some limitations for assessing depression in PwS with the Calgary scale being seen as the preferred measure. However, the DRS is considered a valid measure of depression in complex cases (Vallet et al., 2024). The present study lacked information about negative symptoms and how negative symptoms were related to depression, loneliness and other psychosocial variables measured. It will be important in future studies to include a focus on the relative roles of negative symptoms in relation to depression of OPwS who are lonely (Piejka et al., 2023). Further, in the present study, the diagnosis of schizophrenia was not based on a standardized uniform interview nor was data reported on age of onset or severity of symptoms. Statistical differences found between large samples does not necessarily reflect the clinical experience for each individual person. The predictive power of findings in this study relate holistically to patient groups rather than the individual. Finally, the findings in this study are cross-sectional findings and thus caution is necessary when interpreting these while focussing on causality.

Strengths of the present analysis are the large community-based sample, rather than a sample of convenience potentially providing a better estimate of loneliness. The validity of the interRAI assessment (Nishtala & Jamieson, 2017; Vallet et al., 2024) and the diagnosis of schizophrenia, used to identify OPwS in this study is an official diagnosis based on reports by the participants’ general practitioner or medical records.

## Conclusion

In conclusion, the present analysis supports the hypothesis that ageing with schizophrenia is associated with higher rates of loneliness compared to the general population. It also identifies modifiable correlates of loneliness, in particular, depression, that could be directly addressed through clinical and psychosocial interventions. That is, addressing depressive symptoms in this population may in part be an intervention that will indirectly reduce loneliness in OPwS.
